# Interspecific coordination and intraspecific plasticity of fine root traits in North American temperate tree species

**DOI:** 10.3389/fpls.2013.00242

**Published:** 2013-07-11

**Authors:** Cornelia M. Tobner, Alain Paquette, Christian Messier

**Affiliations:** ^1^Département des Sciences Biologiques, Center for Forest Research, Université du Québec à MontréalMontréal, QC, Canada; ^2^Département des Ressources Naturelles, Institut des Sciences de la Forêt Tempérée, Université du Québec en OutaouaisRipon, QC, Canada

**Keywords:** specific root length, fine root diameter, branching intensity, tree fine roots, phenotypic plasticity, functional traits

## Abstract

Fine roots play an important role in nutrient and water absorption and hence overall tree performance. However, current understanding of the ecological role of belowground traits lags considerably behind those of aboveground traits. In this study, we used data on specific root length (SRL), fine root diameter (D) and branching intensity (BI) of two datasets to examine interspecific trait coordination as well as intraspecific trait variation across ontogenetic stage and soil conditions (i.e., plasticity). The first dataset included saplings of 12 North American temperate tree species grown in monocultures in a common garden experiment to examine interspecific trait coordination. The second dataset included adult and juvenile individuals of four species (present in both datasets) co-occurring in natural forests on contrasting soils (i.e., humid organic, mesic, and xeric podzolic).The three fine root traits investigated were strongly coordinated, with high SRL being related to low D and high BI. Fine root traits and aboveground life-strategies (i.e., relative growth rate) were weakly coordinated and never significant. Intraspecific responses to changes in ontogenetic stage or soil conditions were trait dependent. SRL was significantly higher in juveniles compared to adults for *Abies balsamea* and *Acer rubrum*, but did not vary with soil condition. BI did not vary significantly with either ontogeny or soil conditions, while D was generally significantly lower in juveniles and higher in humid organic soils. D also had the least total variability most of which was due to changes in the environment (plasticity). This study brings support for the emerging evidence for interspecific root trait coordination in trees. It also indicates that intraspecific responses to both ontogeny and soil conditions are trait dependent and less concerted. D appears to be a better indicator of environmental change than SRL and BI.

## Introduction

The search to understand the effects of species on ecosystem functioning has brought forward the functional role of various traits. Functional traits have been shown to link species to the roles they play in the ecosystem. Through changes at the organismal level they not only influence individual performance but also higher organizational levels and hence drive ecosystem processes and services (Diaz et al., 2004; Garnier et al., 2004). However, we know much more about aboveground traits, their coordination, phenotypic plasticity and linkages to ecosystem functioning than we know about belowground traits.

Although the physiological and ecological importance of roots is well established, the great variability of root systems, the small and varied size of fine roots and the relative inaccessibility of the belowground realm have all hampered exhaustive root research. In addition, the lack of consensus about how to classify and measure fine roots has constrained the development of a unified framework toward a root economics spectrum as was achieved for both leaves (Wright et al., 2004) and wood (Chave et al., 2009) traits. Fine roots have traditionally been distinguished from coarser roots using various diameter classes of arbitrary width, with 2 mm being the most common threshold (Pregitzer, 2002; Hishi, 2007; Guo et al., 2008). Consequently, fine root samples of different or even the same species may include varying numbers of root orders. Fine root traits such as specific root length (SRL), diameter, root length density as well as nitrogen, lignin, non-structural carbohydrate, and cellulose concentrations have been found to systematically change with root order (Pregitzer et al., 2002; Guo et al., 2004; Wang et al., 2006). Such morphological and physical changes with root order translate into potentially large differences in functional properties such as water uptake (Rewald et al., 2011), respiration (Jia et al., 2011) or fine root mortality (Wells et al., 2002). More recently, a functional classification approach based on root orders has been applied (Guo et al., 2008; Rewald et al., 2011). In tree roots, a first order root would usually be the smallest (i.e., shortest) segment, which would be attached to a second order branch and so forth (Fitter, 2002). Although this approach attempts to control for confounding factors, comparisons across studies are restricted due to varying numbers of root orders included (see for example Yu et al., 2007; Comas and Eissenstat, 2009; Alvarez-Uria and Körner, 2011; Chen et al., 2013).

Above- and below-ground organs share many functions, such as nutrient acquisition and transfer. Some functional coordination between above and belowground traits is therefore expected (Westoby and Wright, 2006). Despite examples of strong coordination in some traits and ecosystems (Reich et al., 1998; Craine et al., 2001; Tjoelker et al., 2005), results remain inconsistent (Westoby and Wright, 2006; Freschet et al., 2010; Chen et al., 2013).

Apart from mean trait values used to coordinate and characterize species, trait plasticity has gained momentum as a driver of individual fitness and consequently, community dynamics. Evidence is accumulating that through changes in realized niches, trait plasticity can be linked to a species' competitive ability and hence overall fitness (Berg and Ellers, 2010). Due to higher spatial and temporal variability of resources belowground, phenotypic plasticity (i.e., plasticity due to environmental changes) is expected to be greater for below- than aboveground traits. There is also evidence of drastic ontogenetic changes in trait values (Cornelissen et al., 2003) that should be more pronounced in long living organisms such as trees. However, only little information about root acclimations to changes in the environment or in ontogeny is available, especially for trees. In addition, much of our knowledge about plant root function is based on seedling responses (Zobel et al., 2007) and on experiments conducted in pots or containers.

Probably the most studied fine root trait is SRL, the ratio between root length and weight (Zobel et al., 2007). Much like the well-known specific leaf area (SLA) for leaves, SRL is thought to describe the economical aspect of a root by weighing the costs (weight) per potential return (length) (Ryser, 2006). Under the assumption that investment in carbon per unit length should be minimized to exploit a larger volume of soil, SRL is expected to be highly plastic and increase under nutrient limitation. Despite examples confirming the assumption (see Ostonen et al., 2007 for a meta-analysis), increases in SRL with increasing nutrient supply as well as no response to changes in nutrient supply have been reported (see Ryser, 2006 for a summary), with equally variable responses to changes in soil water (Ostonen et al., 2007; Cortina et al., 2008; Bakker et al., 2009).

Through its link to surface area and volume, fine root diameter (D) is an important trait directly linked to nutrient and water absorption. Although D has been shown to be plastic and strongly dependent on nutrient supply (Eissenstat et al., 2000), it is rarely a focus of fine root research except as average diameter (Zobel et al., 2007). Research on the response of D to nutrient concentrations showed species specific responses with increases and decreases possibly depending on nutrient, species and their interaction (Zobel et al., 2007).

Lastly, branching intensity (BI, also called root tip density) is a fine root trait describing the topology of fine roots by counting the number of tips per unit root length. Changes in BI to environmental factors have been assessed in only a handful of studies, with contrasting results (Ahlström et al., 1988; George et al., 1997; Kakei and Clifford, 2002).

In the present study, we examined interspecific (coordination) and intraspecific variation across contrasting soil conditions (i.e., plasticity) as well as with ontogenetic stages (i.e., adults versus juveniles) for SRL, D and BI. A first dataset (“common garden,” CG), including 12 North American temperate tree species grown in a common garden experiment was used to examine trait variation across species. We tested the hypotheses that under uniform controlled conditions:

1. SRL, BI and D are strongly coordinated across species of wide variation in root morphology; and
2. Belowground fine root traits are correlated to whole-plant life-strategies, such as relative growth rate.

A second dataset (“natural forest”, NF) of four tree species (also present in the CG dataset) that included adults and juveniles co-occurring on contrasting soil conditions in natural forests was employed to examine trait variation in relation to species, ontogeny and soil conditions. More specifically, we tested the hypotheses that:

1. SRL and BI are greater and D smaller in juvenile compared to adult trees;
2. SRL and BI generally increase while D decreases with decreasing soil moisture and nutrient content;
3. Phenotypic plasticity is greater in fine root traits that are more strongly associated with resource uptake (i.e., SRL and D).

## Materials and methods

### Common garden dataset—CG

#### Study site

The study site for the first dataset was located at Ste-Anne-de-Bellevue, near Montreal, Québec, Canada (45°26'N, Long 73°56'W, 39 m.s.l). Mean annual temperature is 6.2°C with a mean annual precipitation of 963 mm (climate.weatheroffice.gc.ca). On this former agricultural field that has been managed for several decades (Marc Samoisette, personal communication, October 2011), monocultures of twelve North American temperate forest species were established in spring 2009 with seedlings of 1 (broadleaf) or 2 (conifer) years of age. These monocultures are part of an ongoing experiment on biodiversity and ecosystem functioning with trees (Tobner et al., submitted). Within the objectives of this biodiversity experiment, the 12 species were selected to cover a wide range of functional traits, including angio- and gymnosperms, and early and late successional species: *Acer saccharum* Marsh., *Acer rubrum* L., *Betula alleghaniensis* Britton, *Betula papyrifera* Marsh, and *Quercus rubra* L. as well as seven conifers: *Abies balsamea* (L.) Mill., *Larix laricina* (Du Roi) K. Koch, *Pinus strobus* L., *Pinus resinosa* Aiton, *Picea glauca* (Moench) Voss, *Picea rubens* Sarg., and *Thuja occidentalis* L.

Each species was planted in a square plot of eight by eight individuals (50 × 50 cm). Plots were replicated four times within an area of ~0.6 ha. Plots were weeded manually and a fence was installed to protect against ungulate herbivory.

#### Common garden trait measurements

Traits were measured in September 2011. From each plot, two individuals were selected that were growing in the outer rows (to minimize impacts on the ongoing experiment). This was repeated for each of the four replicate blocks resulting in eight individuals sampled per species. Following the main axis (i.e., stem), a root that grew toward the inside of the plot was detected and followed until it branched off into roots <2 mm. Roots were excavated and placed in a cooler for transport. Roots were then stored at 4°C until processing that occurred no later than 2 weeks after sampling.

Roots were carefully washed and separated into segments of the first three orders. This classification approach (i.e., 1st to 3rd order roots) was chosen following Guo et al. (2008). Root samples were then scanned for subsequent image analysis (Winrhizo, Regent software, Québec). Total root length, average diameter and number of root tips were measured for each sample. Finally, root samples were oven-dried at 65°C and weighed to calculate SRL (m g^−1^). Relative growth rate (RGR) was calculated based on volume ([trunk diameter at 5 cm]^2^ × total tree height): RGR = (log vol fall 2011 - log vol spring 2009)/3 growth periods (i.e., vegetation periods 2009 through 2011).

### Natural forest dataset—NF

#### Study site

The study site for the second dataset was situated at the Station de biologie des Laurentides of Université de Montréal in St-Hippolyte, Québec, Canada (Lat 45°59'N, Long 73°59'W, 366 m.s.l.). The research station consists of an area of about 16 km^2^ of forest and lakes dedicated to research and has been protected from other human activities since 1963. Birch (*Betula papyrifera* and *Betula alleghaniensis*) and maple (*Acer saccharum* and *Acer rubrum*) communities are the dominating forest types covering more than 60% of the land surface in terms of canopy cover (Savage, 2001). Mean annual temperature is 3.9°C with a mean annual precipitation of 1164 mm (climate.weatheroffice.gc.ca).

Four forest species, also present in the CG dataset, co-occur in the forests of the research station on contrasting soil conditions: *Acer rubrum*, *Betula papyrifera*, *Abies balsamea*, and *Thuja occidentalis*. Species were selected to include a broad spectrum of phylogeny and different life strategies (growth rate, life span, type of mycorrhization, etc.). We identified three different soil conditions where the studied species occur:

- Humisols with standing water level between 10 to 20 cm belowground and *T. occidentalis* as the dominant species, hereafter referred to as “humid organic”,
- Orthic humoferric podzols (Courchesne and Hendershot, 1989, personal communication Courchesne, March 2011) on slopes of 28–46° and strong water runoff with *T. occidentalis* as the dominant species, hereafter referred to as “xeric podzol” and
- Orthic humoferric podzols with good drainage, nil to very gentle slope and *B. papyrifera* as the dominant species, hereafter referred to as “mesic podzol.”

For each soil type, three plots covering at least 200 m^2^ were established. Plots were located under closed canopy, with no recent sign of perturbation and at least four adult and four juvenile individuals of the target species. Exceptions were *T. occidentalis* that never occurred on mesic podzols and *B. papyrifera*, for which no juvenile individuals were found, as this species does not regenerate under closed canopies. Juveniles were defined as tree saplings between 25 and 100 cm in height and adult trees were defined as trees with a diameter at 1.3 m (DBH) >10 cm.

#### Soil characterization

At the center of each plot, one soil sample was taken at 20 cm depth on August 22, 2011. The average daily temperature in the 2 weeks preceding soil sampling was 17.5°C. Precipitation for the same period amounted to 46 mm distributed over 6 days with 15 mm being the strongest precipitation event for 1 day.

Soil samples were placed in resealable plastic bags and immediately stored at −18°C before further processing that occurred no later than 1 week after collection. Samples were then oven-dried at 65°C until they reached constant weight and sieved through a 2 mm mesh prior to soil analyses. Soil moisture was the difference in sample weight before and after drying. Soil pH was measured in water in a ratio of one part soil (10 mg) to two parts water for mineral soil and one part soil (4 mg) to five parts water for organic soils (Canadian Society of Soil Sciences, 2007). Cation exchange capacity (CEC) and base saturation (BS%) were assessed through dissolving soil samples in barium chloride solution and atomic spectroscopy (Canadian Society of Soil Sciences, 2007) (Table 1).

**Table 1:** **Soil and stand characteristics of the three soil conditions (means ± sd) for the Natural Forest dataset**.

	**Soil moisture (%)**	**pH**	**CEC (cmol kg^−1^)**	**BS%**	**Basal area (m^2^ha^−1^)**				
					***Abies balsamea***	***Thuja occidentalis***	***Acer rubrum***	***Betula papyrifera***	**others**
HO	85.2 ± 1.8	4.88 ± 1.1	1.9 ± 1.1	95.9 ± 3.4	5.9 ± 2	14.85 ± 2.6	7.1 ± 0.4	6.3 ± 3.5	8.0 ± 4.2
MP	30.7 ± 3.0	5.05 ± 0.0	0.6 ± 0.2	29.9 ± 16.2	7.2 ± 3		4.7 ± 0.4	23 ± 14.1	9.5 ± 5.6
XP	19.2 ± 7.2	4.70 ± 0.3	0.5 ± 0.1	19.1 ± 4.7	6.0 ± 3.4	10.1 ± 5.6	4.0 ± 2.9	6.7 ± 3.0	11.6 ± 14.0

*HO, humid organic; MP, mesic podzol; XP, xeric podzol; CEC, cation exchange capacity; BS, base saturation*.

#### Natural forest trait measurements

On each plot, species and DBH of all adult trees (i.e., DBH >10 cm) were recorded to calculate basal area (Table 1). Adult trees of the site are usually not older than 90 years as the last high-intensity fire passed through the research area around 1923 (Savage, 2001).

For the four target species, at least four adult and four juvenile individuals were sampled (i.e., total of 12 adults and 12 juveniles per soil condition). For each adult tree, two root samples were collected in opposite directions from each other. From the stem, roots were excavated and followed until they branched off into fine roots (<2 mm diameter). Roots of adult individuals were excavated from the mineral or organic soil horizons, never from the humus or litter layers. Furthermore, for each adult individual, at least three of the highest branches were harvested with the help of a professional tree climber to obtain sun leaves. For juveniles, the entire plant was excavated for root samples and at least three leaves or 20 needles were collected.

Leaf and root samples were immediately put into sealed plastic bags, labeled and stored at about 4°C until further processing, occurring no later than 6 weeks after sampling. For each individual, 3–5 leaves were punched with a hollow metal pin, yielding leaf samples of a standard surface area. A minimum of 20 needles of the previous year of growth were plucked off the branch and scanned. Samples were then oven-dried to constant weight to calculate SLA (foliage area/foliage weight, mm mg^−1^).

Root samples (<2 mm) of each individual were carefully washed and scanned and analyzed in an identical fashion to the CG dataset. Once the complete sample was scanned, parts of the image containing first to third order roots were selected and re-analyzed. For these subsamples, average diameter, total length and number of tips were calculated. In addition, root diameter was assessed following the handbook of trait measurements (Cornelissen et al., 2003), on first order roots, after the root hair zone (i.e., after tapering).

Hereafter for both datasets, traits measured on complete root samples (roots <2 mm) are noted using the subscript “c” (e.g., D_c_), while results for fine roots defined as first to third order roots are noted with subscript “3” (e.g., D_3_). Diameter measured on first order roots is noted as “D_1_”.

#### Phenotypic plasticity

The total phenotypic variability of a population is the result of genetic and environmental sources and their interaction (Hartl and Clark, 1997; Whitman and Agrawal, 2009). To quantify the total variability of a trait we employed the coefficient of variance (CV), i.e., the standard deviation divided by the mean.

In a second step, for each trait and species we calculated an index of the variability which is due solely to variation in the environment, the phenotypic plasticity index (PI). Determining the contribution of the environmental source of variability is essential in assessing a population's potential to adapt to heterogeneous or changing environments (Byers, 2008). The ability of a genotype to express different phenotypic values for a given trait under different environmental conditions, the phenotypic plasticity (Valladares et al., 2006), is strongly linked to individual fitness (Bell and Galloway, 2007; Nicotra and Davidson, 2010) and hence population demographics as it can generate novelty and facilitate evolution (Draghi and Whitlock, 2012). Phenotypic plasticity has gained increasing interest with the necessity to predict species responses to global change (Matesanz et al., 2010; Richter et al., 2012). Several metrics have been proposed to assess this environmental source of variability (Valladares et al., 2006). In the present study, we employed the phenotypic plasticity index (PI), a metric recommended to explore functionally related traits. PI is based on maximum and minimum trait means across environmental conditions and was calculated for every trait and species as:

[max(trait mean among soil conditions) − min(trait mean among soil conditions)]/max[trait mean among soil conditions]

(Valladares et al., 2006).

Finally, to compare the phenotypic plasticity with the overall phenotypic variability, we computed a ratio of PI to CV (PI:CV) as an expression of how much of the overall phenotypic variability is due to plastic responses to the environment. Both CV and PI vary between zero and one. Hence, a PI:CV of zero would indicate no environmental source of variability, whereas a PI:CV of one would indicate that the overall phenotypic variability is completely due to acclimations to the environment. Although the literature on trait variation and plasticity is rich, we are not aware of other studies using PI:CV to explore differences in relative plasticity between species and traits.

### Data analysis

For both datasets, traits were tested for normality with the Shapiro test and transformations were applied where needed to correct for deviations. To test for species differences within the CG dataset, a One-Way ANOVA with subsequent Tukey HSD test was performed. Trait correlations were assessed using the Pearson correlation coefficient.

To test for effects of soil condition and ontogenetic stage on fine root traits in the NF dataset, linear mixed effect models (REML) with site (random effect) as well as the interaction of plot and ontogenetic stage nested within soil condition were applied for each species. The asymptotic inference test for coefficients of variation as described in Miller and Feltz (1997) was used to test for differences in CV as well as PI:CV between traits and species. Subsequent Dunn-Sidak corrections (Šidák, 1967) were applied to correct alpha levels for multiple comparisons. To test for differences in PI, resampling methods were applied to create populations per species, ontogenetic stage and trait (*N* = 999). Data were then analyzed using ANOVA models to test for effects of trait and species.

## Results

### Interspecific trait coordination (CG)

In the common garden, fine root traits were highly coordinated across species, especially SRL_3_ and D_3_ (Table 2). SRL_3_ increased with BI_3_ and decreased with D_3_. Consequently, BI_3_ was negatively correlated with D_3_. Correlations between fine root traits and whole plant strategies such as RGR were much weaker and never significant (Table 2). In general, conifers showed greater D_3_, lower SRL_3_, and BI_3_ (Table 3).

**Table 2:** **Correlation matrix for functional traits of 12 North American temperate forest species grown in a common garden**.

	**D_3_**	**SRL_3_**	**BI_3_**
SRL_3_	**−0.83**		
BI_3_	**−0.64**	**0.66**	
RGR	0.05	0.07	0.07

*Traits include belowground specific root length (SRL), diameter (D) and branching intensity (BI) as well as whole-plant life-strategy measures (i.e., relative growth rate – RGR). Fine root traits were measured on first three root orders (subscript “3”). Significant correlations appear in bold type (*P*<0.001 in all cases)*.

**Table 3:**
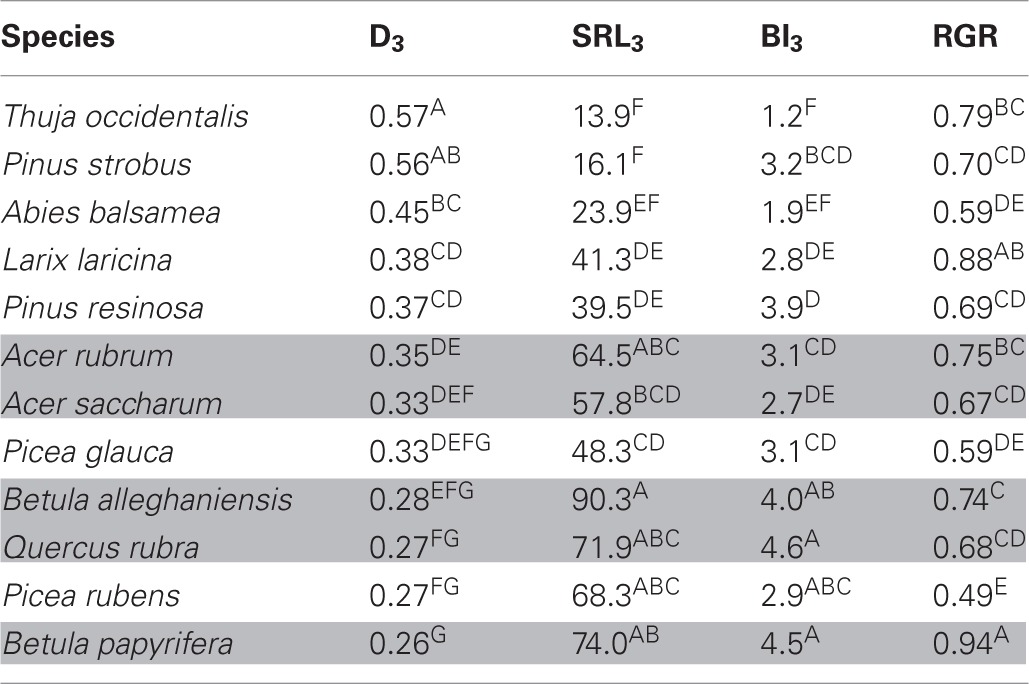
**Mean trait values for 12 North-American temperate forest species grown in a common garden**.

*Traits include belowground specific root length (SRL), diameter (D) and branching intensity (BI) as well as whole-plant life-strategy measures (i.e., relative growth rate—RGR). Fine root traits were measured on first three root orders (subscript “3”). Different letters indicate significant differences between species. Angiosperms are underlined in gray*.

### Intraspecific trait variation across ontogenetic stages and contrasting soil conditions (NF)

**Figure 1:**
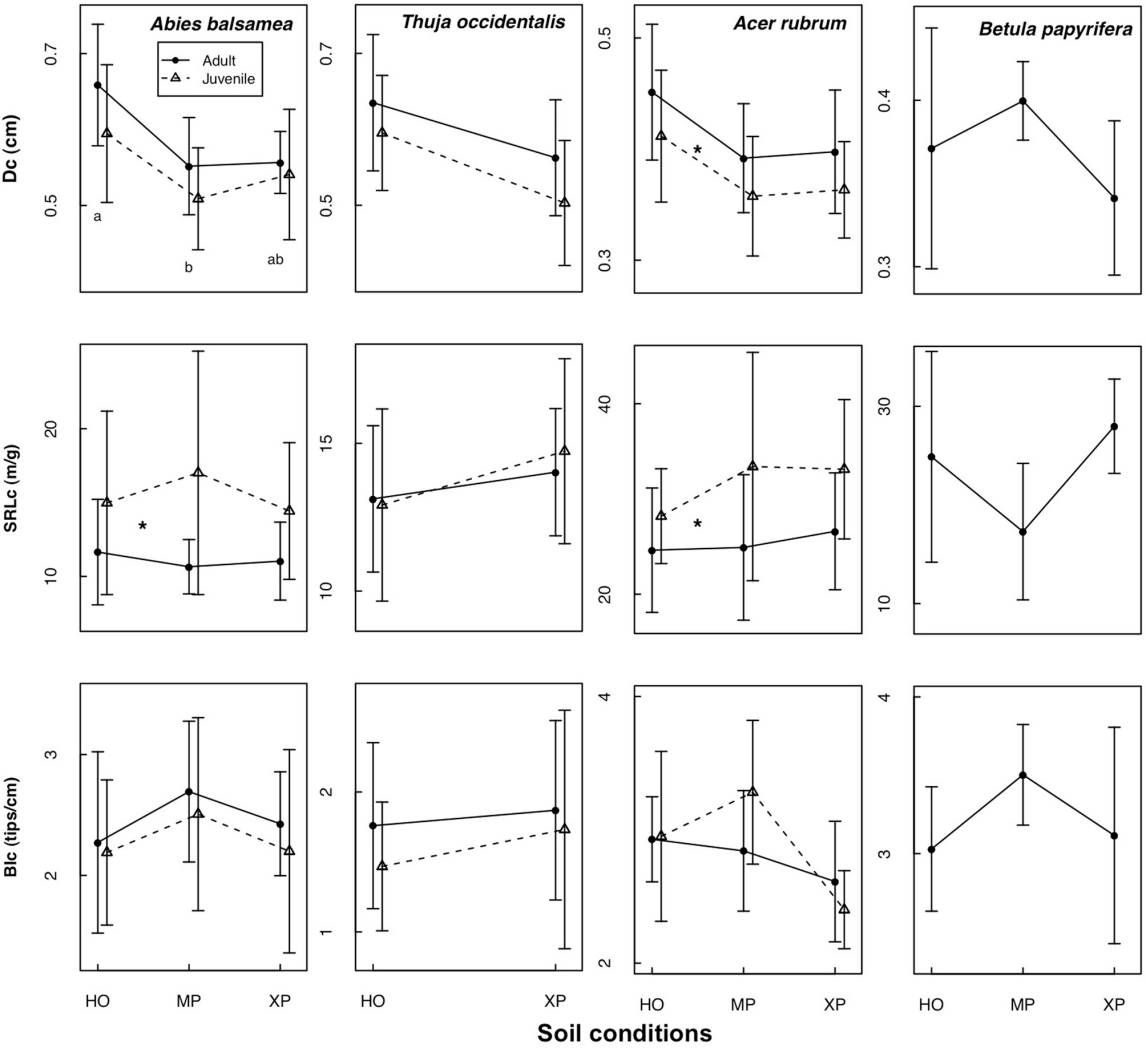
**Mean ± standard deviation for three fine root traits along a gradient of soil conditions (NF dataset).** Traits are measured on roots <2 mm: specific root length (SRL_c_), branching intensity (BI_c_) and fine root diameter (D_c_). Soil conditions were identified as HO, humid organic; MP, mesic podzol; and XP, xeric podzol. Different letters indicate significant differences between soil conditions; asterisks indicate significant differences between adults (solid line) and juveniles (dashed line) (for *P* < 0.05).

In the natural forest, fine root diameter in woody (i.e., D_c_ and D_3_) as well as non-woody roots (i.e., D_1_) was generally greater in humid organic than in mesic and xeric podzol conditions. However, differences were only significant for *A. balsamea* and *T. occidentalis* (Tables 4, 5). D was also significantly lower for juveniles compared to adults in all three species (Tables 4, 5 and Figure 1). While differences for *A. rubrum* were consistent across fine root classification (i.e., size versus functional) for *T. occidentalis* differences were only significant for the two functional classifications of fine roots (i.e., D_3_ and D_1_), and for *A. balsamea* there only were significant differences in non-woody roots (i.e., D_1_, Tables 4, 5).

**Table 4:** ***P*-values for fixed effects (soil condition and ontogenetic stage—OS) of linear mixed models (REML) and their interactions on functional traits of four North-American temperate forest species (NF dataset)**.

		**Diameter**			**SRL_c_**	**Branching intensity**		**SLA**
		**D_c_**	**D_3_**	**D_1_**		**BI_c_**	**BI_3_**	
*Abies balsamea*	Soil	0.03^*^	0.07^•^	<0.01^**^	0.95	0.67	0.17	0.47
	OS	0.12	0.30	0.03^*^	0.01^*^	0.58	0.44	<0.01^**^
	Soil+OS	0.72	0.43	0.20	0.71	0.98	0.96	0.34
*Thuja occidentalis*	Soil	0.09^•^	0.02^*^	0.03^*^	0.22	0.77	0.60	0.66
	OS	0.09^•^	0.02^*^	<0.01^**^	0.72	0.21	0.71	<0.01^**^
	Soil+OS	0.71	0.95	0.67	0.51	0.66	0.59	0.42
*Acer rubrum*	Soil	0.13	0.76	0.14	0.55	0.11	0.10	0.09^•^
	OS	0.04^*^	0.04^*^	<0.01^**^	0.02^*^	0.63	0.13	<0.01^**^
	Soil+OS	0.99	0.33	0.53	0.75	0.33	0.47	0.04^*^
*Betula papyrifera*^1^	Soil	0.15	0.54	0.10	0.15	0.50	0.65	0.77

*Traits include belowground specific root length (SRL), diameter (D) and branching intensity (BI) as well as aboveground specific leaf area (SLA). Fine root traits were measured on roots <2 mm (subscript “c”), first three root orders (subscript “3”) or first order roots only (subscript “1”)*.

Significant effects are annotated as

** *P < 0.01*,

* P < 0.05, and

• *P < 0.1*.

1 *No B. papyrifera juveniles were found in the NF plots*.

**Table 5:** **Mean/coefficient of variance (CV) for three fine root traits measured on the same root samples but following different fine root classification approaches**.

**Species**	**OS**	**Soil condition**	**Diameter**			**Specific root length**	**Branching Intensity**	
			**D_c_**	**D_3_**	**D_1_**	**SRL_c_**	**BI_c_**	**BI_3_**
*Abies balsamea*	A	HO	**a** 0.66/0.12	0.62/0.11	**a** 0.53/0.18	11.7/0.31	2.3/0.33	2.1/0.39
		MP	**b** 0.55/0.12	0.46/0.23	**b** 0.39/0.14	10.7/0.17	2.7/0.22	2.7/0.23
		XP	**b** 0.56/0.07	0.55/0.15	**a** 0.48/0.16	11.0/0.24	2.4/0.18	2.0/0.30
		All sites	0.59/0.14	0.55/0.20	0.47/0.20	11.1/0.25 **AB**	2.5/0.25 **AB**	2.3/0.32
	J	HO	**a** 0.60/0.15	0.55/0.19	**a** 0.45/0.12	15.0/0.41	2.2/0.27	2.2/0.34
		MP	**b** 0.51/0.13	0.47/0.15	**ab** 0.40/0.15	17.0/0.49	2.5/0.32	2.8/0.22
		XP	**ab** 0.54/0.16	0.50/0.13	**b** 0.39/0.12	14.4/0.32	2.2/0.38	2.2/0.28
		All sites	0.55/0.16	0.51/0.17	0.42/0.14	15.5/0.42 **a**	2.3/0.32 **ab**	2.4/0.29
*Thuja occidentalis*	A	HO	0.64/0.14	**a** 0.65/0.10	**a** 0.60/0.05	13.1/0.19	1.8/0.34	1.4/0.24
		XP	0.56/0.14	**b** 0.55/0.21	**b** 0.51/0.13	14.0/0.15	1.9/0.34	1.3/0.24
		All sites	0.60/0.15	0.60/0.18	0.55/0.14	13.6/0.17 **B**	1.8/0.33 **A**	1.3/0.24
	J	HO	0.59/0.13	**a** 0.57/0.13	**a** 0.51/0.09	12.9/0.25	1.5/0.31	1.6/0.37
		XP	0.50/0.16	**b** 0.46/0.13	**b** 0.40/0.18	14.7/0.21	1.7/0.49	1.4/0.42
		All sites	0.55/0.17	0.52/0.16	0.46/0.18	13.8/0.24 **b**	1.6/0.43 **a**	1.5/0.39
*Acer rubrum*	A	HO	0.45/0.14	0.40/0.14	0.42/0.13	24.6/0.27	2.9/0.11	3.6/0.21
		MP	0.39/0.13	0.36/0.17	0.36/0.19	24.9/0.31	2.8/0.16	3.6/0.24
		XP	0.40/0.14	0.39/0.17	0.36/0.14	26.6/0.23	2.6/0.17	3.1/0.28
		All sites	0.41/0.15	0.39/0.16	0.38/0.17	25.4/0.26 **AB**	2.8/0.15 **b**	3.4/0.25
	J	HO	0.41/0.14	0.32/0.18	0.36/0.11	28.2/0.18	3.0/0.22	3.0/0.23
		MP	0.36/0.15	0.33/0.14	0.31/0.15	33.4/0.36	3.3/0.16	3.5/0.21
		XP	0.36/0.12	0.35/0.13	0.34/0.14	33.1/0.22	2.4/0.12	2.7/0.25
		All sites	0.38/0.15	0.33/0.15	0.34/0.14	31.3/0.28 **b**	3.0/0.21 **BC**	3.1/0.24
*Betula papyrifera*	A	HO	0.37/0.20	0.29/0.32	0.30/0.16 **b**	24.9/0.43	3.1/0.13	3.7/0.29
		MP	0.40/0.06	0.26/0.35	0.22/0.26 **a**	17.3/0.40	3.5/0.09	3.8/0.20
		XP	0.34/0.14	0.26/0.21	0.23/0.17 **b**	28.0/0.17	3.1/0.22	4.1/0.23
		All sites	0.36/0.16	0.27/0.29	0.25/0.24	23.2/0.38 **A**	3.2/0.17 **B**	3.9/0.24

*Subscript “c” indicates a trait measured on roots <2 mm, subscript “3” indicates a trait measured on first to third order roots and subscript “1” indicates a trait measured on first order roots (diameter only). Data shown separately according to ontogenetic stage (OS): A, Adults; J, Juveniles and soil conditions; HO, Humid organic; MP, Mesic podzol and XP, Xeric podzol soil conditions. Different letters to the left of a column indicate significant differences in mean; different letters to the right of a column indicate significant differences in CV between soil conditions. Letters for all sites indicate significant differences between species (upper case for adults, lower case for juveniles)*.

SRL_c_ never varied significantly across soil conditions but was significantly greater for juveniles compared to adults in *A. balsamea* and *A. rubrum*. For juveniles of *T. occidentalis*, SRL_c_ was smaller as well, but did not vary significantly (Tables 4, 5 and Figure 1). Conversely, BI_c_ never varied significantly across soil conditions or ontogenetic stage (Tables 4, 5).

**Figure 2:**
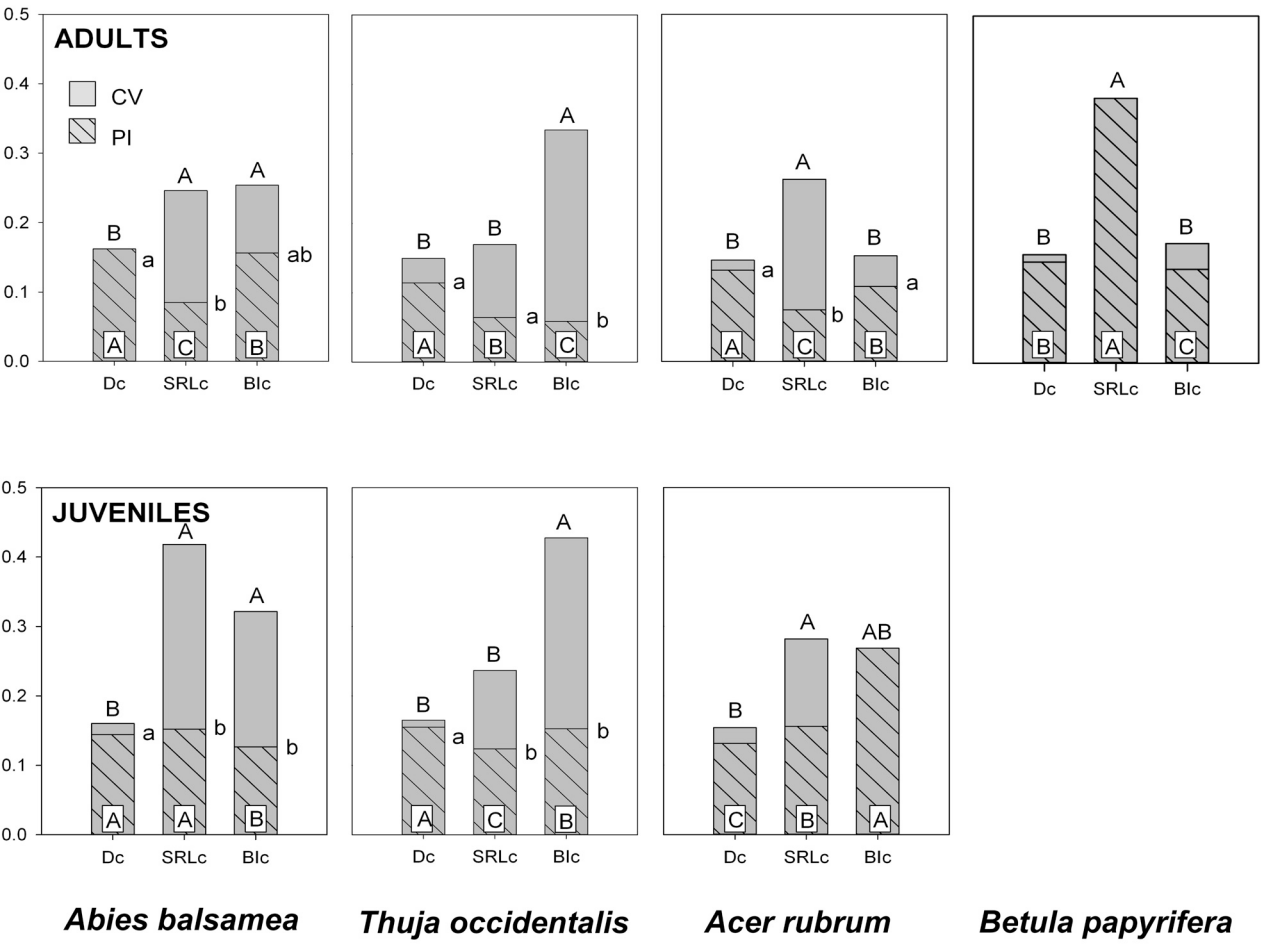
**Coefficient of variation (CV, gray) and the phenotypic plasticity index (PI, gray hatched) for fine root traits of four North-American temperate forest species (NF dataset).** Different letters indicate significant differences between traits (capital letters for CV, capital letters on white inset for PI and small letters for PI:CV). Traits include specific root length (SRL_c_), branching intensity (BI_c_) and fine root diameter (D_c_) and were measured on roots <2 mm (subscript ‘c’). Trait effects for CV and PI:CV were computed using the asymptotic interference test (Miller and Feltz, 1997). Trait effects for PI were computed on resampled populations and consecutive ANOVA models.

PI was greatest in D_c_ except for *B. papyrifera* adults and *A. rubrum* juveniles. PI for SRL_c_ and BI_c_ was more variable and depended on species (Figure 2). The amount of total trait variability (CV), tended to be significantly higher in SRL_c_ and BI_c_, compared to D_c_ (Figure 2). Consequently, D_c_ was also the trait with the highest PI:CV.

As expected, SLA was significantly higher in shade-grown leaves of juveniles compared to sun leaves of adults (Table 4). SLA did not vary significantly with soil conditions. The significant interaction term of soil condition and ontogenetic stage for *A. rubrum* is due to a slightly higher SLA for juveniles in mesic conditions (Table 4). When analyzed by species and ontogenetic stage, no significant correlation was found between SLA and SRL (data not shown).

Although fine root classification based on root orders did not uniformly reduce variation (i.e., CV) compared to fine root classification based on size (Table 5), in some cases, it helped detect treatment differences (e.g., D_c_ to D_3_ for *T. occidentalis*, Tables 4, 5).

## Discussion

### Interspecific trait coordination

The observed belowground trait correlations across various taxa indicate strong coordination among fine root morphological traits supporting the idea of a generalized tree root syndrome (Holdaway et al., 2011).

As root diameter and root mass density constitute the two components of SRL, the strong negative correlation between SRL and D was expected (Fahey and Hughes, 1994; Comas and Eissenstat, 2009; Chen et al., 2013). Branching patterns were found to negatively correlate with D when measured as BI (i.e., number of root tips divided by root length, Comas and Eissenstat, 2009) or as branching ratio (number of root tips divided by number of second order roots, Chen et al., 2013) and positively with SRL (Comas and Eissenstat, 2009). As shown by Comas and Eissenstat (2009), there is a possible link between BI and mycorrhization that may in turn determine internal cell structure (e.g., layers of root cortex) and hence D and SRL.

Although evidence is still sketchy, root syndromes are based on a trade-off between life-history strategies (e.g., RGR) and tissue longevity. Thus, roots with high SRL, thin D and low tissue density are generally associated with greater root proliferation, greater RGR and shorter overall longevity (Eissenstat, 1992; Wright and Westoby, 1999). In previous studies, growth rates of juvenile and adult trees have been linked to root traits with fast-growing species showing higher SRL (Reich et al., 1998; Comas et al., 2002; Comas and Eissenstat, 2004), smaller root diameter and greater degree of branching (Comas et al., 2002; Comas and Eissenstat, 2004, note that for these papers, results are for phylogenetically constrained contrasts). Other studies documented no or even negative relationships between SRL and SLA or RGR in grasslands (Poorter and Remkes, 1990; Laughlin et al., 2010; Kembel and Cahill, 2011) and trees (with phylogenetic independent contrasts, Chen et al., 2013).

In the present study, no significant relationships were found between fine root traits and RGR based on volume, height or diameter (only volume is reported). Here, the two species with highest SRL were also the species with the highest and lowest RGR (*B. papyrifera* and *P. rubens*, respectively). The study site for the common garden experiment has been intensively cultivated for decades. Nutrient availability can be assumed to be abundant. Interestingly, the four species occurring in both datasets have markedly higher SRL (less so for *T. occidentalis*) in the common garden site, compared to the nutrient poorer natural forest, confuting the often-assumed increase in SRL with nutrient limitation. This indicates that in nutrient abundant habitat, SRL may not be a trait of primary importance for plant growth.

### Trait variation between ontogenetic stages

Trait responses to ontogenetic stage were trait dependent. Similar trends of decreasing SRL with age as shown in our study have been reported in the literature for Japanese cedar (*C. japonica*) (Fujimaki et al., 2007), silver birch (*B. pendula*) (Rosenvald et al., 2012), European spruce (*P. abies*) and Turkey oak (*Q. cerris*) (Claus and George, 2005) or in a comparison of laboratory-grown seedlings to field-grown adult trees of six temperate North American tree species (Comas and Eissenstat, 2004). D was also found to increase with tree age (Jagodziński and Kaucka, 2010; Rosenvald et al., 2012).

Two possible mechanisms may explain differences in root morphology with age. On the one hand, higher SRL and lower D in juveniles could be an artifact of differences in root orders measured as it is likely that juvenile root samples <2 mm contain fewer root orders than their conspecific adults. For a multitude of species, SRL and D have been shown to significantly change with root order (Pregitzer et al., 2002; Wang et al., 2006). However, when controlling for root orders in both adults and juveniles, SRL was still higher in juveniles compared to adult trees (Comas and Eissenstat, 2004; Rosenvald et al., 2012).

It appears thus more likely, that the observed changes in root morphology with ontogenetic stage may be an adaptation to rooting depth. In most of the above-mentioned studies examining the effect of tree age on root morphology, including the present study, soil depth was not accounted for. However, changes in SRL and diameter with soil depth have been reported in other studies (Wang et al., 2006; Makita et al., 2011). In the present study, root samples for adult trees were collected in the mineral horizons (often below 10 cm soil depth) while the entire root system of juveniles often did not exceed 10 cm soil depth. Furthermore, juveniles were frequently found on or near rotting logs. Increased SRL and lower D of juveniles could thus be an acclimation to shallow soil depth and possible higher nutrient availability. This is congruent with the assumption that species experiencing large shifts in height and therefore environmental conditions while maturing should experience corresponding shifts in traits (Grime, 2001; Smilauerova and Smilauer, 2007).

It was surprising that BI never changed significantly with ontogenetic stage. In fact, BI also never changed significantly with soil condition, pointing toward a rather conservative trait and fine root topology.

### Trait plasticity across soil conditions

As shown above with ontogenetic stages, fine root responses to soil conditions were also trait specific. Despite the large gradient in soil nutrients and water (Table 1), SRL and BI never varied significantly across soil conditions for the four target tree species; only D tended to be greater in humid organic soils.

SRL has been studied extensively and it was often associated with root proliferation in response to nutrient heterogeneity (Hodge, 2004). For trees, SRL has even been described as a successful indicator of nutrient availability (Ostonen et al., 2007). Empirical responses of SRL to increases in nutrients have been mixed, however, (Ryser, 2006). Initially, it was proposed that under growth limiting conditions, SRL should be greater (and D smaller) in order to decrease construction costs and invest in greater soil exploitation (Ryser, 2006). And indeed, decreases in SRL with nutrients have been documented (Trubat et al., 2006; Ostonen et al., 2007). However, positive (Majdi and Viebke, 2004; Yu et al., 2007) or non-significant (George et al., 1997; Mei et al., 2010) responses of SRL to nutrients have been documented as well. Despite advances in root research, responses of SRL to nutrient availability still appear somewhat “mysterious” (Ryser, 2006) and SRL has been shown to vary significantly with type of fertilizer, sampling method (i.e., pot, soil coring or ingrowth core) and root diameter class sampled (i.e., 0–1 mm, <2 mm, etc.) (Ostonen et al., 2007).

As mentioned earlier, SRL has two components: diameter and root mass density. While SRL did not change significantly with soil conditions, D was higher in humid organic conditions compared to mesic and xeric podzolic conditions implying a possible inverse response of root mass density that could explain the lost signal in SRL. In grasses, decreases in nitrogen and phosphorus have been shown to decrease root diameter and increase tissue mass density (Ryser and Lambers, 1995). If the same applied to temperate tree species, then humid organic conditions with their greater water and nutrient content (Table 1) would constitute an improvement in plant nutrition. Tissue density in roots has been related to the proportion of stele and of cell wall in the stele, and to characteristics of the tracheary system (Wahl and Ryser, 2000). A reduced percentage of stele in fine roots with decreasing tissue mass density could indicate a reduced importance of conductive tissue in an environment of good plant nutrition as in humid organic soil conditions. Although some studies have reported increases in D with nutrients (Holdaway et al., 2011) and water (Peek et al., 2005; Cortina et al., 2008), its potential as environmental indicator may have been underestimated so far.

A limited number of studies have examined responses of BI to soil nutrition, reporting mostly non-significant changes (George et al., 1997; Bakker et al., 2000). Interestingly, among these few studies on BI, contrasting results were reported within species (i.e., *Pinus sylvestris*) (Ahlström et al., 1988; George et al., 1997). In the present study, BI proved to be the least variable and least plastic fine root trait responding to neither ontogenetic stage nor soil conditions.

### Trait plasticity

From the three fine root traits assessed in the present study, D clearly showed the greatest plasticity (PI) and was also the trait where phenotypic plasticity contributed the most to total phenotypic variability (highest PI:CV). This coincides with it being the most responsive trait to soil conditions (Tables 4, 5). Although more often used to assess acclimations to changes in the environment, SRL_c_ had significantly greater CV and a lower PI:CV than D_c_ in most cases. Interestingly, the species with the greatest CV within SRL_c_ are the two ectomycorrhizal species, *A. balsamea* (juvenile) and *B. papyrifera* (Table 5 and Figure 2), indicating that this greater variability may be due in part to methodological challenges of hyphenated root samples.

Variability of BI was highly species specific. In adults and juveniles, CV for BI_c_ was similar to those of D_c_ for the two angiosperm species and significantly higher for the two gymnosperm species. In addition, CV was generally higher in juveniles compared to adults. This trend is reversed in many cases when measured on D_3_, D_1_, or BI_3_ (Table 5), indicating a possible effect of greater variation in root orders comprised in samples <2 mm for juveniles.

## Conclusion

Fine root morphological traits were found to be strongly coordinated across species, but further work is needed to test for general patterns across ecosystems and biomes. Above- and below-ground traits and whole-plant-strategies may not be as coordinated as previously thought once other factors such as site productivity are accounted for or controlled as we have done in this study for the common garden experiment. For the natural forest experiment, fine root traits responded differently to soil conditions within species, with fine root diameter being the most responsive. Diameter showed the least total variation yet much of it was explained by changes in the environment. Consequently, D may be the most suitable trait for evaluating plasticity to soil nutrition for the rhizosphere.

Lastly, the present study underscores the need for a unified framework of fine root classification and stronger control for the many possible confounding factors in root studies. Although a functional classification of fine roots managed to reduce variance in a limited number of cases, it improved estimator evaluation in at least one species. Most importantly, a unified framework would greatly facilitate the comparison of studies and therefore increase current understanding of the functional ecology of roots.

### Conflict of interest statement

The authors declare that the research was conducted in the absence of any commercial or financial relationships that could be construed as a potential conflict of interest.
